# Enhancement of Filtration Performance Characteristics of Glass Fiber-Based Filter Media, Part 1: Mechanical Modification with Electrospun Nanofibers

**DOI:** 10.3390/ma17102209

**Published:** 2024-05-08

**Authors:** Laura Weiter, Stephan Leyer, John K. Duchowski

**Affiliations:** 1 Faculty of Science, Technology and Communication, University of Luxembourg, 4365 Luxembourg, Luxembourg; laura.weiter@hydac.com (L.W.); stephan.leyer@uni.lu (S.L.); 2 HYDAC FluidCareCenter® GmbH, 66280 Sulzbach, Germany

**Keywords:** physical modification, nonwoven filter media, electrospun nanofibers, pore size control

## Abstract

Various modifications of standard glass fiber filtration media using electrospun PA66 nanofibers are described. PA66 were selected because they were readily available from commercial sources. Other polymers, such as PP, PET and PBT, could also be used. The first set of samples was prepared by mixing the nanofibers at two, three and five weight percent with glass fibers, and the second by laying the same proportion of the nanofibers directly onto the downstream side of the substrate. The aim of these modifications was to improve the three most basic functionalities of filter media, the separation efficiency, the differential pressure (ΔP) and the dirt holding capacity (DHC). The modified media samples were evaluated with the standard textile characterization techniques and filtration performance evaluation procedures. The results showed differences in the several tens of percentage points achieved with the two modification methods. Moreover, additional differences in performance were observed depending on the percentage of nanofibers admixed to the substrate. These differences were most apparent in the filtration efficiency and the DHC, both by several percentage points, with no apparent effect on the ∆P. The results strongly suggest that the preparation of new filter media by incorporating nanofibers directly into the matrix can result in significant improvements in filtration performance characteristics.

## 1. Introduction

The most common materials used in the preparation of fibrous matrices employed in the usual industrial filtration applications include cellulose, glass fibers and various synthetic polymers. The fibers can either be wet laid onto a substrate or directly blown onto a belt where they form a self-contained layer [1,2,3]. Collectively, these are referred to as nonwoven filter media as the fiber orientation within the material is totally random except for the certain amount of directionality imparted to it by the paper machine during the wet laying process. The introduction of glass fibers as the main material for the preparation of filter media sometime in the 1960s represented a breakthrough in the achievable filtration performance in comparison to the previously mostly employed cellulose [4]. This was a result of much finer fiber diameters, more “convoluted” or “twisted” fiber orientation within the fiber matrix both of which created many more tortuous flow channels within the media allowing for more efficient particle capture without restricting the flow. Although a reproduction of similar structures was attempted with the extruded synthetic polymeric melt blown materials, it was possible to achieve the same performance characteristics due to the generally larger fiber diameters [5,6,7].

**Figure 1:**
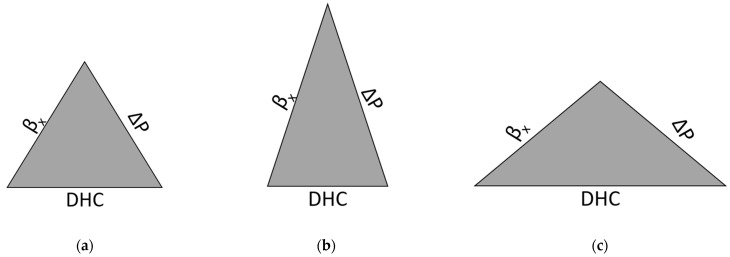
(**a**) Representation of the beta ratio, ΔP and the DHC triangle. For example (**b**), a higher beta ratio and a higher ∆P result in a smaller DHC; and for example (**c**), a smaller beta ratio and a smaller ∆P result in a higher DHC.

Nonwoven glass fiber filter media constitute the most basic ingredient employed in filter element construction of nearly all industrial functional fluids [8]. They exhibit excellent performance characteristics in terms of all three fundamental properties, namely the filtration efficiency, typically reported as the beta ratio (βx), differential pressure (ΔP) and the dirt holding capacity (DHC) that make them far superior to other materials such as the nonwoven polymer based meltblowns [8,9,10]. The continued drive towards further improvement of the glass fiber media performance characteristics typically involves the optimization of the three aforementioned parameters [11]. This is usually accomplished by adjusting the mixture of glass fibers from which the overall fiber matrix is prepared by mixing fibers of different diameters and lengths [12]. The diameters of coarse structures usually range from approximately 10 μm down to 1 μm, whereas the lengths are typically on the order of several millimeters [13]. The unfortunate drawback of this approach is that by tugging on one apex of the beta ratio, ΔP and the DHC triangle as shown in Figure 1 pulls the other two apices in, such that an increase in the filtration efficiency almost invariably results in the increase in pressure drop and a concomitant loss of the DHC [2,14,15,16].

Nevertheless, the work on this approach continues as the sources of fibers of even smaller diameters (down to the nanometer range) become increasingly available without a significant penalty in material costs. The aim of this investigation was therefore to incorporate fibers with diameters well below 500 nm and explore their impact on the filtration performance characteristics illustrated in Figure 1 above.

**Figure 2:**
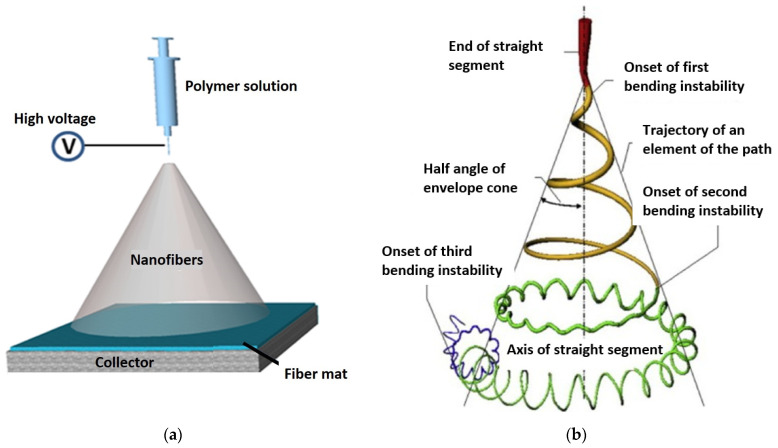
(**a**) Setup to produce nanofibers. (Reprinted with permission from [21]. Copyright {2024} American Chemical Society). (**b**) Path of an electrospinning jet that contained three successive electrical bending instabilities [22,23]. (Reprinted with permission from [22]. Copyright {2024} American Chemical Society).

A relatively new type of development in the preparation of filter media that contain smaller diameter fibers is to employ the electrospinning process [17]. The electrospinning process is a method of producing nanofibers (NF) by applying an electric field to a polymer solution. During the process, the polymer solution is extruded through a nozzle, drawn into fine fibers by the electric field as shown in Figure 2. This method is often used in materials science to give materials special properties [18,19,20]. These special properties include but are not limited to the fiber matrix stiffness, pore size, amount of void volume, etc., such that they impart enhanced filtration performance characteristics previously not attainable with the standard filtration media without modification.

Polymer nanofibers produced by electrospinning can be utilized to produce novel structures such as membranes with various different properties such as the extremely high specific surface area, high porosity (typically 90%), and light weight [24]. This modification is based on the fact that the NF are spun onto one side of the basic filter substrate where they form an additional layer. This changes the physical structure of the filter material. 

Although the physical change in structure increases the retention efficiency of the base substrate, this means that a conventional nonwoven filter medium loses its depth filtration properties. Standard depth filter media exhibit high dirt holding capacities, as the contaminants accumulate in the depth of the medium and the entire filter media volume can therefore be used for contaminant capture. If there is an additional polymer nanofiber layer on the upstream side of the substrate, it forms a fine barrier to which the particles adhere and the volume of the filter material cannot be utilized to its full potential. This effect is required in specific applications, such as air filtration, where it is employed to achieve backwash ability. To backwash a filter medium, the volume flow is reversed. This removes most of the contamination accumulated on the surface. This process increases the service life of a filter [7,25,26,27].

**Figure 3:**
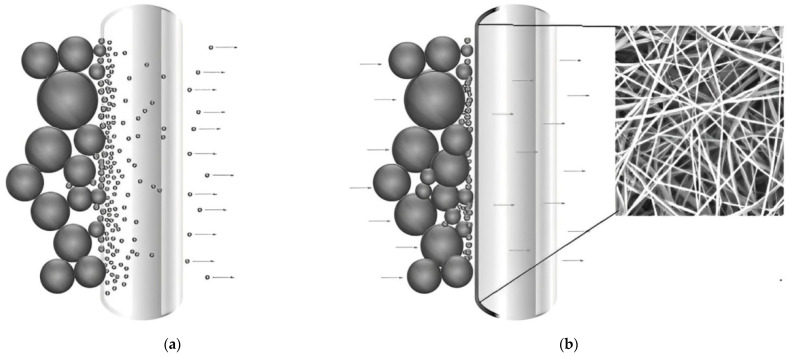
(**a**) Depth filtration and (**b**) surface filtration with electrospun nanofibers [28]. The arrows in the figure represent the direction of the volume flow.

If the polymer nanofiber layer is on the downstream side, the base material can be used to full advantage until exhausted; however, the downstream layer of the nanofibers creates an additional barrier to fluid flow that results in a significant higher ∆P. Once a filter has reached a defined terminal differential pressure, for example of 3 bar or 6 bar depending on the application, the filter element needs to be replaced. The differences in overall functionality achieved with the depth (a) versus surface filtration (b) based on the different use of the NF and the substrate are illustrated graphically in Figure 3 below.

However, it should be noted that a polymer nanofiber layer increases the differential pressure, whether on the upstream or downstream side. As a result, electrospun nanofibers are used in air filtration applications or in applications where the fluid viscosities or where the flow rates are low. For example, in typical hydraulic applications, the flow rates or rather fluid fluxes are typically on the order of 0.04 L min^−1^ cm^−2^ or somewhat higher. Taken together with fluid viscosities >32 mm^2^ s^−1^, this would result in an unacceptably high ∆P across the filter element.

In this paper, we compare and contrast the performance characteristics of new filter media prepared with electrospun NF incorporated directly into the glass fiber matrix against that with the NF layer deposited on the downstream side. The main goal of this approach was to see if relatively simple modifications to the standard fiber matrix could be made to enhance the three properties of interest enumerated in Figure 1. That is instead of formulating an entirely new recipe for the main substrate, which is usually the case, the intent was to see if by admixing a certain, relatively low percentage of the NF into the substrate or depositing a thin layer of the NF on the surface would bring about sufficient and positive changes to the filtration performance characteristics without involving major machine runs that the development of new filtration substrates usually require.

## 2. Materials and Methods

The prepared filter media samples are usually evaluated to determine their so-called primary properties, such as the length to width ratio, tenacity, flexibility, and cohesiveness, as well as their secondary properties, such as morphology, physical shape and specific gravity. However, for the purposes of this paper, we have limited ourselves to evaluating those properties that are related or govern the process of filtration itself. The methodologies employed for this purpose are described directly below.

### 2.1. Textile Material Characterization Procedures

The prepared filter media samples were evaluated by the standard textile characterization methods that included thickness, basis weight, air permeability [11] and the mean flow pore size (MFP) [29]. These procedures are summarized in Table 1 below [11,30,31,32,33,34].

### 2.2. Evaluation of the Filtration Performance Characteristics

The impact of the physical fiber matrix modifications was determined in accordance with the standard ISO Test procedures, namely, ISO 16889:2022 [39], “Hydraulic fluid power—Filters–Multi-pass method for evaluating filtration performance of a filter element” and ISO 3968:2017 [40], “Hydraulic fluid power–Filters–Evaluation of differential pressure versus flow” [41] for differential pressure measurements depending on the volume flow Q (DPQ). For the multipass evaluation, a circular media sample with an effective filtration area of 176.71 cm^2^ was employed. The test was carried out with a flow rate of 7 L min^−1^ and the base upstream gravimetric (BUG) content of 8.12 mg L^−1^ of ISO Medium Test Dust (ISO MTD). The test stand employed the standard MIL-H-5606 fluid and operated at a temperature of 40 °C at which the fluid exhibits a viscosity of 15 mm^2^ s^−1^. The test was terminated with the differential pressure across the filter sample reached 3 bar. The pressure loss characteristics of the prepared filter media samples were evaluated at the viscosity of 30 mm^2^ s^−1^ to eliminate any nonlinearity effects. The 30 mm^2^ s^−1^ evaluation was performed with Megol HLP ISO VG 32 fluid at respective temperature of 40 °C. The flow rate was stepped from rest to 10 L min^−1^ in as small stepping intervals as possible (approx. 0.005 L min^−1^) or until the terminal differential pressure of 5 bar was reached.

### 2.3. The Impact of Physical Filter Media Modifications

**Figure 4:**
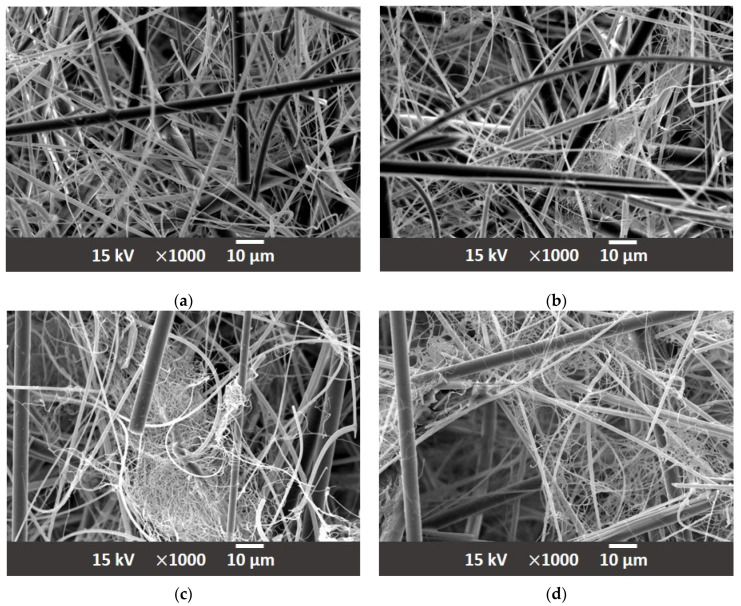
(**a**) Substrate A; (**b**) A-2 with 2% PA66 nanofiber content; (**c**) A-3 with 3% PA66 nanofiber content; and (**d**) A-5 with 5% PA66 nanofiber content.

**Figure 5:**
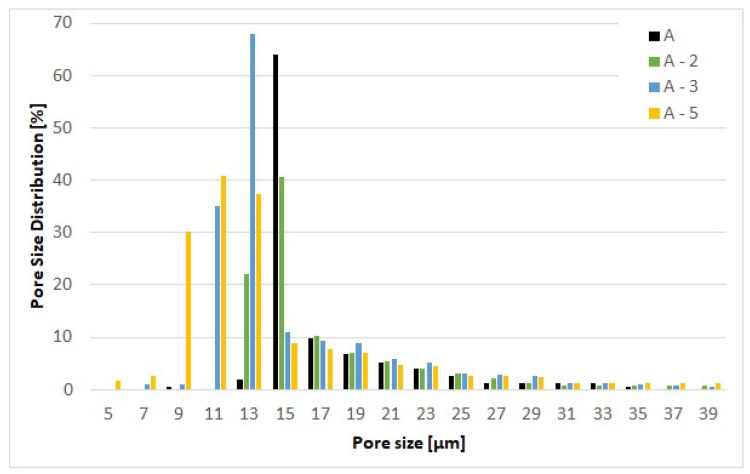
Pore size distribution of the base substrate A and the mechanical modifications A-2, A-3 and A-5.

The first approach employed in preparing modified filter media samples was based on the inner fiber matrix modification. This involved admixing PA66 electrospun nanofibers with diameters down to several hundred nanometers at varying proportions to the main fiber matrix. In contrast to the standard, typical coarse fiber diameters of approx. 1 µm to 10 µm, the range of fiber diameters in the newly prepared matrix was extended down to 100 nm. The PA66 nanofibers were obtained from a commercial source Tong Li Tech Co. Ltd., Shenzhen, China. The nanofibers were incorporated directly into the glass fiber matrix in the usual way the glass fiber filter media are prepared. They were mixed in a water solution with an added acrylate binder and wet laid onto sieve. Water was then removed by vacuum and the prepared filter medium oven dried. The PA66 NF were chosen for their ready commercial availability; however, other polymers, notably, PET, PBT, PEO, PS, PMMA, PVP, PAA, PVA, PU and PCL could be equally well used, providing they mix well with the glass fibers in the substrate and exhibit appropriate chemical compatibility for the application [42,43,44,45]. The samples in which the nanofibers were incorporated into the substrate were produced using a sheet forming system from Estanit GmbH in accordance with the DIN EN ISO 5269-2 [46]. Three modified formulations were prepared, the first contained 2% (A-2) polymer nanofiber content, second 3% (A-3) and third 5% (A-5) of nanofiber content. A sample composition of thusly prepared nonwoven materials is shown in the scanning electron micrographs in Figure 4, at a magnification of ×1000. The resulting impact of the carried out fiber matrix modifications is summarized in Table 2. The corresponding mean flow pore size distribution of the filter media is reported in Figure 5.

**Figure 6:**
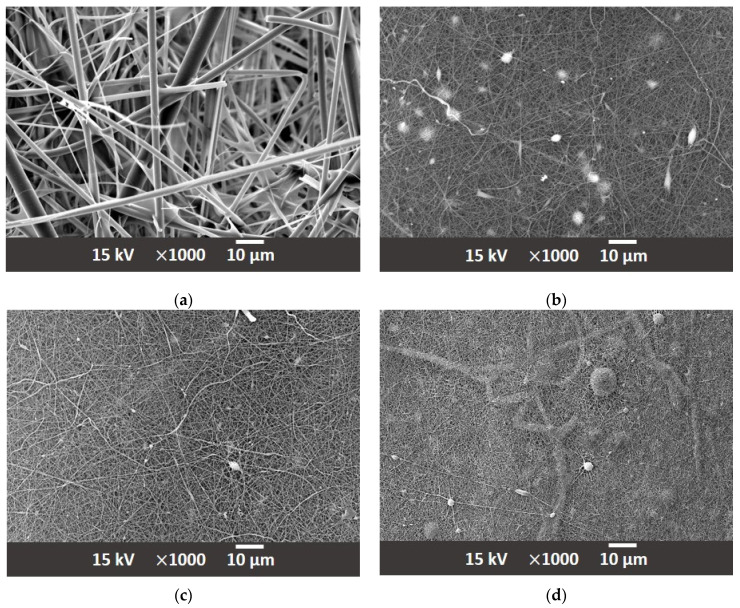
(**a**) Substrate B; (**b**) B-2; (**c**) B-3; and (**d**) B-5.

**Figure 7:**
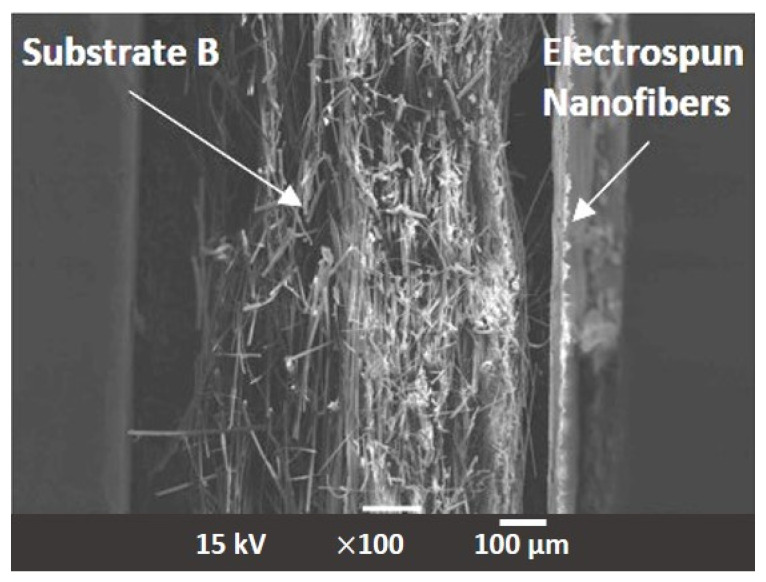
Side view of the electrospun PA66 nanofibers on the substrate B. Shown here as an example is B-2.

**Figure 8:**
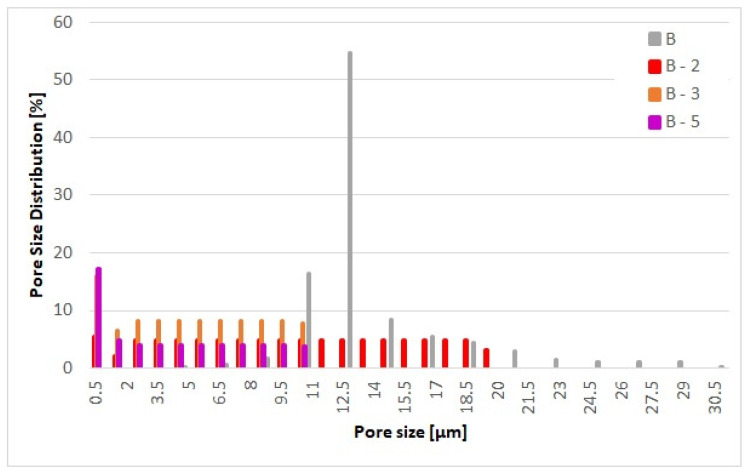
Pore size distribution of the base substrate B and the modifications B-2, B-3 and B-5.

In addition to the in-matrix formulation, the effect of the on-substrate formulation was also evaluated with the NF in the same proportion to the glass fiber employed in the in-matrix formulation. The production of the on-substrate material required the use of the base material be supplied in rolls. The rolls are necessary to ensure the uniform distribution of the NF layer during the deposition process. If a static process were used (Figure 2a), the nanofibers would accumulate in the middle of the substrate and the homogeneity of the second layer would not be guaranteed. Nonwoven materials, especially the glass fiber media, always have an upstream and downstream side that determines their direction of use in an application. The downstream side is more compact due to the manufacturing process of the nonwoven media. In order to maintain the effect of depth filtration and not generate surface filtration, the nanofibers were deposited on the downstream side [26]. Figure 6 shows the SEM images the appearance of the downstream side of the individual filter media where the NF are located. Figure 7 shows a sideview of the filter medium B-2 and clearly demonstrates how the electrospun nanofiber layer acts as an additional layer on the downstream side of the substrate. Table 3 shows the physical properties of the base substrate B with its three modifications. The first modification contains 2% (B-2), the second 3% (B-3) and the third 5% (B-5) nanofibers in relation to the base substrate weight. The changes in pore size distribution can be seen in Figure 8 for all four filter media.

## 3. Experimental Results

Three experiments were carried out for each filter medium, the mean values and corresponding standard deviations of which are shown below.

**Figure 9:**
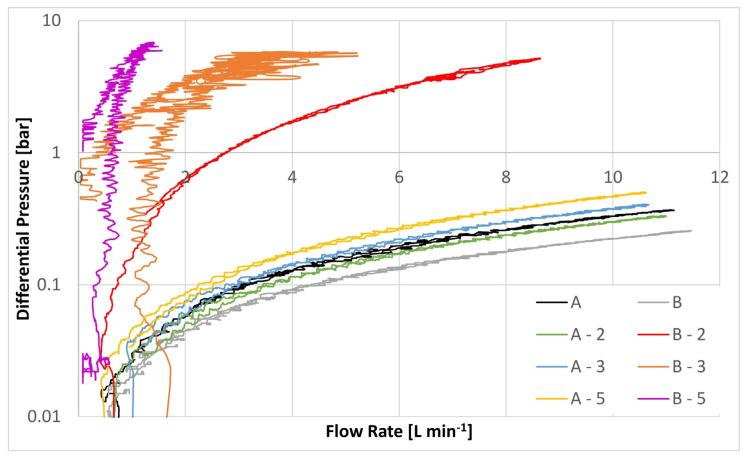
∆P results for the mechanically modified nonwovens.

Figure 9 shows the dependence of the differential pressure on the volume flow. All base substrates and all modifications were evaluated with Megol HLP 32, which has a viscosity of at 30 mm^2^ s^−1^ at 40°. Figure 9 shows that the differential pressure at 1 L min^−1^ is already 2 bar for modification B-3 and 5 bar for modification B-5. 

**Figure 10:**
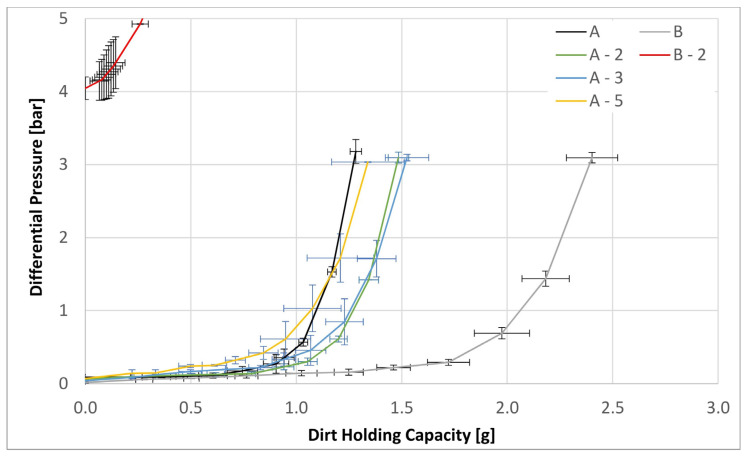
The dirt holding capacity for the both base substrates A and B as well as the modifications A-2, A-3, A-5 and B-2.

Figure 10 does include the result for the B-2 on-surface modification to show how far away it lies from all three in-matrix modifications. Because the B-3 and B-5 would be over further outside of the experimental window they discarded from further considerations.

**Figure 11:**
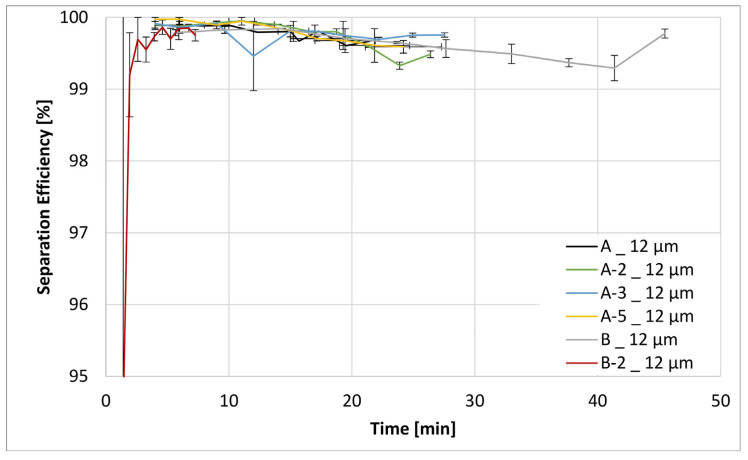
Separation efficiencies for the both base substrates A and B as well as the modifications A-2, A-3, A-5 and B-2 for a contamination particle size > 12 µm.

**Figure 12:**
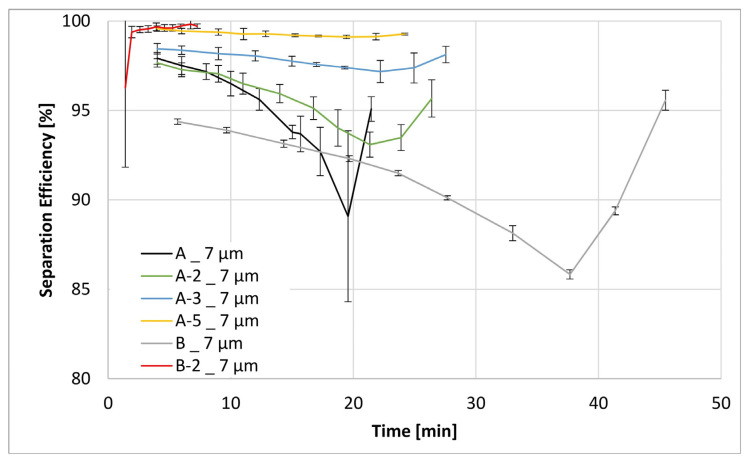
Separation efficiencies for the both base substrates A and B as well as the modifications A-2, A-3, A-5 and B-2 for a contamination particle size > 7 µm.

**Figure 13:**
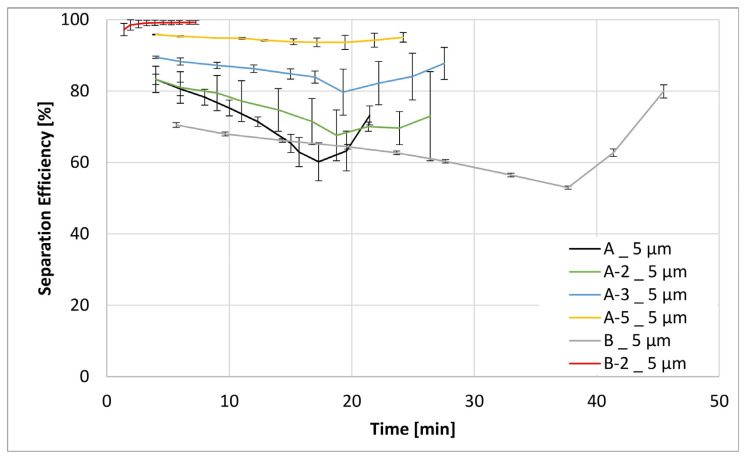
Separation efficiencies for the both base substrates A and B as well as the modifications A-2, A-3, A-5 and B-2 for a contamination particle size > 5 µm.

**Figure 14:**
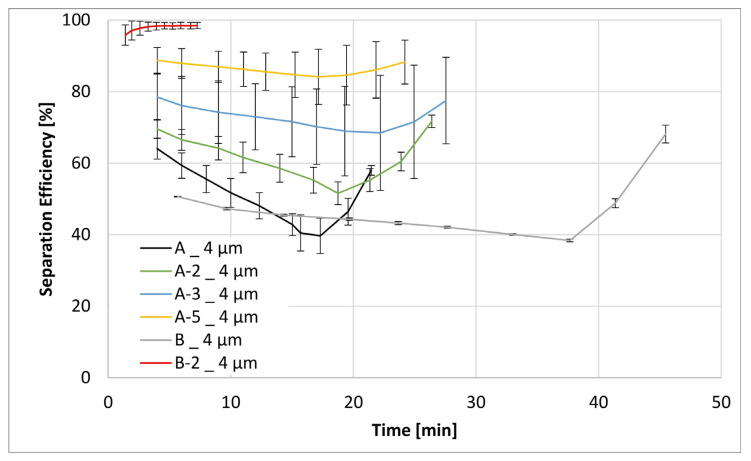
Separation efficiencies for the both base substrates A and B as well as the modifications A-2, A-3, A-5 and B-2 for a contamination particle size > 4 µm.

Figure 11, Figure 12, Figure 13 and Figure 14 below show the separation efficiency for different contamination particle sizes for the respective formulations of different filter media. The particle sizes are divided into >12 µm, >7 µm, >5 µm and >4 µm. The separation efficiencies were derived from the ISO 16889:2022 [39] test where the following expression applies:

(1) $$SE=\frac{N_{b}-N_{a}}{N_{b}}\;\cdot 100$$

- N_b_ = Number of particles before the filter medium;
- N_a_ = Number of particles after the filter medium.

## 4. Discussion

The determination of the physical properties such as thickness and basis weight primarily provide a description of filter media in terms of its most basic physical properties. If these values are comparable for two different media samples, other properties such as the air permeability and mean flow pore size can be used for further assessment. The latter two parameters provide a more complete description of what can be expected in terms of filtration performance characteristics from the prepared and/or modified media samples. The air permeability reflects the expected differential pressure, whereas the mean flow pore size is indicative of the expected separation efficiency.

The polymer nanofibers were introduced directly into the A substrate. By introducing polymer nanofibers into the substrate, the internal structure of the matrix is changed. However, this depends on the proportion of the nanofibers added. This is illustrated in Table 2 that shows that a 2% nanofiber content has no effect on the air permeability and the MFP value compared to the base material without nanofibers. At this small nanofiber fraction, two filter media are readily comparable. However, already at 3% or 5% and more so at 5% nanofiber fraction a marked reduction in the air permeability and MFP value are observed. This means that the material becomes denser and therefore finer on the inside. The reduction in the MFP is readily confirmed at 5% NF content as material A-5 exhibit on MFP value of 2.89 µm for A-5, in contrast to 4.48 µm of the original substrate. The relative pore size distributions of the filter media with varying NF content are compared in Figure 4, in which a shift to finer MFP values with increasing NF content is clearly observed.

In comparison, Table 3 shows considerable differences between the basic substrate B and the filter media with the additional surface deposited nanofiber layer. The 2% content already causes a huge reduction in the air permeability value by 200 L m^−2^ s^−1^. A very interesting and quite unexpected behavior can be observed for the MFP values of the materials prepared with surface deposited nanofibers. This is summarized in Table 3 which lists the textile properties of the filter media prepared with surface deposited nanofiber fractions. For example, at 2% content virtually no change in the MFP value is observed. However, there is a considerable shift in both the maximum and the minimum pore size, respectively. In particular, the maximum pore size has been reduced from 30 µm down to 22 µm (27%) and on even greater shift from 5 µm to 0.6 µm (88%) has been observed for the minimum pore size. At a higher nanofiber fraction content, a concomitant reduction in both the MFP and the maximum pore size is also observed; however, the minimum pore size appears to have reacted an asymptotic value of 0.6 µm.

Taken collectively the textile properties of the two differently modified substrates, i.e., those with the nanofibers admixed directly into the fiber matrix and those deposited on the surface show huge differences in their respective fundamental behavior. The collected data suggest that admixing the nanofibers directly into the matrix brings about an increase in the number of internal flow channels as illustrated by the relatively minor changes in air permeability and the MFP. In contrast, by depositing the nanofibers on the surface provides an additional and rather defense barrier to flow that dramatically effects both air permeability and the MFP.

The above textile property behavior is likewise reflected by filtration performance characteristics derived from the macro scale DPQ and multipass tests.

The effects of the barrier-like properties of the nanofibers as an additional layer become clear in the experiments. In this regard, Figure 9 shows that all three modifications in which the nanofibers were deposited on the substrate exhibited an enormous increase in the differential pressure as a function of flow rate in contrast to the original base material. Here, the original filter medium B showed a ∆P of 0.019 bar at a flow rate of 1 L min^−1^, whereas modified media exhibited ∆Ps of 0.155 bar, 2 bar, and 5 bar for the B-2, B-2 and the B-5 variants, respectively (cf. Table 4, DPQ). In contrast, with the same gravimetric content of the nanofibers admixed into the fiber matrix, it was possible to reach flow rates of up to 8 L min^−1^ without reaching excessively high DPQs. Specifically, whereas the original material (A) exhibited a ∆P of 0.262 bar, the modified version exhibited comparable values of 0.239 bar, 0.3 bar and 0.367 bar for the A-2, A-3 and A-5 modifications, respectively.

The excessively high ∆Ps of the B-3 and B-5 surface laid modifications precluded the evaluation of either the DHC or filtration efficiencies for these variants because the multipass test needed to operate at a minimum flow rate of 7 L min^−1^. Only the B-2 variant could be evaluated on the multipass test stand and that by allowing the ∆P to reach 5 bar instead of the usual 3 bar by form of exception. Nevertheless, a DHC of only 0.25 g was reached by 5 bar, thereby testing a significantly denser matrix structure described above. In contrast, the original material B reached a DHC value of 2.4 g and that already by the terminal ∆P of 3 bar.

In contrast, the in-matrix nanofiber materials behaved in a strikingly different manner to their on-surface counter parts. In particular, all modifications exhibited a higher DHC compared to the parent material as listed in Table 4. Even at a highest fraction of 5%, the modified material exhibited a DHC value nearly 5% higher compared to the original material, with the A-2 and A-3 variants exhibiting corresponding values of nearly 16% and 19%, respectively. This is a fact worth noting, especially in view of the fact that all three modification exhibited reduced MFP values indicative that at successive admixtures into the matrix, the material became progressively finer. These observations strongly suggest that even through the internal flow channels became smaller with higher nanofiber content, their numbers must have increased thereby resulting in a higher DHC.

These findings are likewise reflected in the results of filtration efficiency evaluations, as shown in Figure 11, Figure 12, Figure 13 and Figure 14. The differences in filtration performance characteristics among all filtration materials start to become readily apparent when the particle size of the test contaminant (ISO MTD) drops below the intrinsic porosity of the prepared fiber matrix, that is below 12 µm. This is especially apparent in Figure 12, Figure 13 and Figure 14. Evaluation of the separation efficiency trends depicted in these three figures clearly shows that the on-substrate B-2 modification is an outlier in the picture. In contrast, trends in increasing filtration efficiencies that are well matched to the fractional content of nanofibers within the matrix. Moreover, this trend is readily apparent and carries for all particle sizes present within the test contaminant. Similarly, it is also consistent with the textile data obtained earlier, most notably the MFP and the max/min pore size values.

Some words of explanation about the convex shape of the filtration efficiency curves are required. We observe similar effects in our typical multipass evaluations of both the flat sheet materials as well as the assembled filter elements. Although it is not possible to observe this directly, the most likely explanation is that it has to do with the displacement and/or rearrangement of the sandy contaminant cake that forms on the surface of the test element. This displacement results in a partial contaminant release from the filter and therefore manifests itself as a dip in separation efficiency. As illustrated in the figures, this effect is temporary; and after a while, the system recovers to its original equilibrium condition. 

## 5. Conclusions

The results reported in the present paper strongly suggest that significant improvements in filter performance characteristics can be realized through judicious manipulation of fiber type and content in prepared fiber matrices. This is particularly true in the case of direct incorporation of polymeric nanofibers directly into the glass fiber substrate. What is most promising about the results reported herein is that the limitation of the three-property triangle appears to have been overcome. As the results have shown, it was possible to realize significant gains in filtration efficiencies and pressure drops without the undue penalties in dirt holding capacities. Although the present investigation has been somewhat limited in terms of the fiber type and content, it nevertheless clearly demonstrated that new and as yet uncharted fiber matrix formulations may bring about even better results. As the availability of the various nanofiber materials increases with a concomitant reduction in their cost, there are possibilities for new and more imaginative filter media formulations, including those that are as yet difficult to realize for economic reasons. Moreover, in this paper, we did not explore other potential applications, such as air and/or water filtration apart from determining the most fundamental textile properties such as air permeability and mean flow pore size. Nevertheless, the rather excellent agreement between those most fundamental textile properties and the filtration performance characteristics exhibited on a macro scale are certainly indicative that a brand new path for such investigations into the science of filtration materials has now been paved.

## Figures and Tables

**Table 1:** Overview of the physical properties to be defined with the corresponding standard method and used apparatus.

Physical Properties	Method	Apparatus
Thickness	DIN EN ISO 5084:1996 [35]	Thickness gauge without pressure—Mitutoyo Deutschland GmbH, Neuss, Germany
Basis Weight	DIN EN 12127:1997 [36]	MSA225P-000-DA—Sartorius AG, Göttingen, Germany
Air Permeability	DIN EN ISO 9237:1995 [37]	FX3300—Textest AG, Schwerzenbach, Switzerland
Pore Size Distribution	ASTM F316-03:2011 [38]	Capillary Flow Porometer AX 1100—Porous Materials INC., Ithaca, NY, USA

**Table 2:** Determination of physical properties of the modification A, where the polymer nanofibers are processed into the substrate structure with 2% (A-2), 3% (A-3) and 5% (A-5) nanofiber content.

Samples	Thickness [mm]	Basis Weight [g m^−2^]	Air Permeability [L m^−2^ s^−1^]	Min Pore [µm]	Max Pore [µm]	MFP [µm]
A	0.5 ± 0.0	75.5 ± 1.8	230 ± 7	4.5 ± 0.0	35.0 ± 0.1	14.9 ± 0.3
A-2	0.5 ± 0.0	76.2 ± 3.2	230 ± 11	4.6 ± 0.1	42.0 ± 1.1	14.8 ± 0.0
A-3	0.5 ± 0.0	76.7 ± 0.7	185 ± 5	3.6 ± 0.1	40.5 ± 3.2	12.7 ± 0.2
A-5	0.5 ± 0.1	72.4 ± 8.8	152 ± 1	2.9 ± 0.1	36.7 ± 7.5	10.4 ± 1.4

**Table 3:** Determination of physical properties of the modification B, where the polymer nanofibers are on the substrate with 2% (B-2), 3% (B-3) and 5% (B-5) nanofiber content.

Samples	Thickness [mm]	Basis Weight [g m^−2^]	Air Permeability [L m^−2^ s^−1^]	Min Pore [µm]	Max Pore [µm]	MFP [µm]
B	0.5 ± 0.0	71.7 ± 1.7	230 ± 22	5.1 ± 0.2	30.3 ± 0.1	12.8 ± 0.5
B-2	0.5 ± 0.0	70.5 ± 1.1	30 ± 3	0.6 ± 0.0	22.3 ± 4.8	12.3 ± 2.4
B-3	0.5 ± 0.0	69.6 ± 2.6	27 ± 9	0.6 ± 0.0	11.8 ± 0.7	6.6 ± 1.0
B-5	0.6 ± 0.0	67.8 ± 2.4	20 ± 9	0.6 ± 0.0	8.4 ± 2.8	6.3 ± 1.4

**Table 4:** Results in mean values for all experiments for both base substrates and all modifications.

Sample	Separation Efficiency [%] for Particle Size [µm]				DHC [g]		DPQ [bar]		
	>4	>5	>7	>12	3 bar	5 bar	1 L min^−1^	2 L min^−1^	8 L min^−1^
A	50.6	71.4	94.9	99.8	1.28		0.03		0.26
A-2	61.5	74.7	95.6	99.8	1.48		0.03		0.24
A-3	73.0	85.5	97.9	99.8	1.52		0.02		0.30
A-5	86.3	94.5	99.3	99.8	1.34		0.04		0.37
B	46.8	64.4	91.4	99.6	2.40		0.02		0.18
B-2	97.9	98.9	99.3	99.2		0.25	0.16		4.56
B-3							2	3.36	
B-5							5		

## Data Availability

The original contributions presented in the study are included in the article, further inquiries can be directed to the corresponding author/s.
